# Novel SMAC-mimetics synergistically stimulate melanoma cell death in combination with TRAIL and Bortezomib

**DOI:** 10.1038/sj.bjc.6605687

**Published:** 2010-05-11

**Authors:** D Lecis, C Drago, L Manzoni, P Seneci, C Scolastico, E Mastrangelo, M Bolognesi, A Anichini, H Kashkar, H Walczak, D Delia

**Affiliations:** 1 Department of Experimental Oncology, Fondazione IRCCS Istituto Nazionale Tumori, Via G Venezian 1, 20133 Milano, Italy; 2 Centro Interdisciplinare di Studi Biomolecolari e Applicazioni Industriali (CISI), Università degli Studi di Milano, Via Fantoli 16/15, 20138 Milano, Italy; 3 Istituto di Scienze e Tecnologie Molecolari (ISTM), Consiglio Nazionale delle Ricerche (CNR), Via Fantoli 16/15, 20138 Milano, Italy; 4 Dipartimento di Chimica Organica e Industriale, Università degli Studi di Milano, Via Venezian 21, 20133 Milano, Italy; 5 Dipartimento di Scienze Biomolecolari e Biotecnologie e CNR-INFM, Università degli Studi di Milano, Via Celoria 26, 20133 Milano, Italy; 6 CNR-INFM S3, National Research Center on Nanostructure and BioSystems at Surfaces, Via Campi 213/A, 41100-Modena, Italy; 7 Institute for Medical Microbiology, Immunology and Hygiene, University of Cologne, Goldenfelsstrasse 19-21, 50935 Köln, Germany; 8 Department of Medicine, Tumour Immunology Unit, Imperial College London, Hammersmith Hospital Campus, 10th floor, Commonwealth Building, Du Cane Road, London W12 0NN, UK

**Keywords:** SMAC-mimetics, TRAIL, apoptosis, melanoma

## Abstract

**Background:**

XIAP (X-linked inhibitor of apoptosis protein) is an anti-apoptotic protein exerting its activity by binding and suppressing caspases. As XIAP is overexpressed in several tumours, in which it apparently contributes to chemoresistance, and because its activity *in vivo* is antagonised by second mitochondria-derived activator of caspase (SMAC)/direct inhibitor of apoptosis-binding protein with low pI, small molecules mimicking SMAC (so called SMAC-mimetics) can potentially overcome tumour resistance by promoting apoptosis.

**Methods:**

Three homodimeric compounds were synthesised tethering a monomeric SMAC-mimetic with different linkers and their affinity binding for the baculoviral inhibitor repeats domains of XIAP measured by fluorescent polarisation assay. The apoptotic activity of these molecules, alone or in combination with tumour necrosis factor-related apoptosis-inducing ligand (TRAIL) and/or Bortezomib, was tested in melanoma cell lines by MTT viability assays and western blot analysis of activated caspases.

**Results:**

We show that in melanoma cell lines, which are typically resistant to chemotherapeutic agents, XIAP knock-down sensitises cells to TRAIL treatment *in vitro*, also favouring the accumulation of cleaved caspase-8. We also describe a new series of 4-substituted azabicyclo[5.3.0]alkane monomeric and dimeric SMAC-mimetics that target various members of the IAP family and powerfully synergise at submicromolar concentrations with TRAIL in inducing cell death. Finally, we show that the simultaneous administration of newly developed SMAC-mimetics with Bortezomib potently triggers apoptosis in a melanoma cell line resistant to the combined effect of SMAC-mimetics and TRAIL.

**Conclusion:**

Hence, the newly developed SMAC-mimetics effectively synergise with TRAIL and Bortezomib in inducing cell death. These findings warrant further preclinical studies *in vivo* to verify the anticancer effectiveness of the combination of these agents.

Members of the IAP (inhibitor of apoptosis protein) family are key regulators of apoptosis and of clinical prognostic value in cancer (Kempkensteffen *et al*, 2007). Together with cellular IAP (cIAP) 1 and 2, XIAP (X-linked IAP) is one of the three most studied IAPs. The structure of XIAP is characterised by three N-terminal tandem repeat domains, called BIRs (baculoviral inhibitor repeats), peculiar of the IAP family, and a C-terminal RING (really interesting new gene) finger, endowed with E3 ubiquitin-ligase function. XIAP is a major negative regulator of apoptosis, a property dependent on its BIR domains (Takahashi *et al*, 1998) that bind to and inhibit both initiator caspase-9 and effector caspases 3 and 7 (Deveraux *et al*, 1997). cIAP1 and cIAP2, also bind caspases (Eckelman and Salvesen, 2006) but the biological significance of this interaction is less well understood. XIAP can regulate caspases and other proteins, not only by binding and sequestering them, but also by ubiquitination and consequent proteasome-dependent degradation.

The activity of XIAP is antagonised by SMAC/DIABLO (second mitochondria-derived activator of caspase/direct inhibitor of apoptosis-binding protein with low pI), that, on release from mitochondria in response to apoptotic stimuli (Du *et al*, 2000), undergoes maturation and cleavage of its N-terminal region, allowing the exposure of the AVPI sequence. This tetrapeptide binds XIAP (Wu *et al*, 2000) and competes with the same binding sites that are responsible for the interaction with caspases (Liu *et al*, 2000). In this way, SMAC/DIABLO prevents the sequestration of caspases, facilitating the apoptotic pathway. As the AVPI sequence can promote apoptosis, compounds mimicking this tetrapeptide, called SMAC-mimetics, have become the focus of intense research (Li *et al*, 2004; Oost *et al*, 2004; Mastrangelo *et al*, 2008) as pro-apoptotic pharmacological agents for cancer treatment (Sun *et al*, 2006). Accordingly, several XIAP-targeting SMAC-mimetics have been developed. Intriguingly, they were also found to target other members of the IAP family, especially cIAP1 and cIAP2 (Varfolomeev *et al*, 2007; Vince *et al*, 2007; Galbán *et al*, 2009). To improve their activity, dimeric SMAC-mimetics formed by two binding heads tethered by a chemical linker, were recently generated and shown to be more potent than the monomeric counterparts (Li *et al*, 2004; Lu *et al*, 2008) because of the simultaneous binding to two BIR domains (Cossu *et al*, 2009b).

In some cell lines, SMAC-mimetics as single agents elicit cell death through the extrinsic apoptotic pathway (Gaither *et al*, 2007) triggered by autocrine production of tumour necrosis factor (TNF) (Petersen *et al*, 2007; Varfolomeev *et al*, 2007; Vince *et al*, 2007; Wang *et al*, 2008) and requiring caspase-8 activation (Bertrand *et al*, 2008). In the vast majority of cancer cells, however, SMAC-mimetics as single agents are poorly cytotoxic (Petersen *et al*, 2007), although they can potently synergise with the apoptosis-inducing ligands of the TNF superfamily (Li *et al*, 2004). Although TNF and CD95 ligand (CD95L/FasL) are unsuitable for cancer treatment because of their systemic inflammatory effects (Lejeune *et al*, 1998) and hepatotoxicity (Walczak *et al*, 1999), respectively, the TNF-related apoptosis-inducing ligand (TRAIL) is an extremely promising biotherapeutic, owing to its potency and limited side effects (Ashkenazi and Herbst, 2008). In spite of the fact that 50% of tumour cell lines are TRAIL-sensitive, it was recently shown that primary tumour cells derived from human breast, lung and colon tumours are often TRAIL-resistant (Todaro *et al*, 2008). Although TRAIL has been suggested for treatment of melanoma (Kurbanov *et al*, 2005), a tumour typically resistant to chemotherapy, also melanomas are often unresponsive to TRAIL-induced apoptosis (Kurbanov *et al*, 2006). Therefore, it is important to identify drugs, which when used in combination with TRAIL, can overcome this resistance.

In this study, we show that human melanoma cell lines can be sensitised to TRAIL *in vitro* by knocking down XIAP by shRNA interference. Furthermore, Bortezomib, a proteasome inhibitor currently used in cancer treatment, can also sensitise melanoma cells to TRAIL. Finally, we describe new SMAC-mimetic compounds, synthesised on the basis of structure-based approaches that markedly synergise with TRAIL in killing melanoma cells.

## Materials and methods

### Cell culture and reagents

All the cell lines (Me1007, Me4405, Me10538, Me2211M2, MDA-MB231) were maintained in RPMI plus 10% fetal calf serum. Bortezomib (Velcade) was kindly provided by Professor Carlostella, isoleucine zipper (iz)-TRAIL was produced as described (Ganten *et al*, 2006). SMAC-mimetics were synthesised at the CISI Institute. The primary antibodies were purchased from Cell Signaling Technology (Beverly, MA, USA) (cleaved caspase-3 and cleaved PARP), Alexis Biochemicals (San Diego, CA, USA) (cFLIP, caspase-8), BD Biosciences (San Jose, CA, USA) (XIAP and cIAP2), Sigma (St Louis, MO, USA) (*β*-Actin) and R&D Systems (Minneapolis, MN, USA) (cIAP1). ECL-HRP linked secondary antibodies were from GE Healthcare (Piscataway, NJ, USA). siCASP8 SMARTpool was purchased from Dharmacon (Lafayette, CO, USA), z-vad-fmk from BIOMOL Int (Plymouth Meeting, PA, USA).

### Cell proliferation and apoptosis

Cell proliferation was determined by the MTT assays on cells seeded in 96-well culture plates (10 000 cells per well) with 100 *μ*l of medium, treated 24 h later with drugs and incubated for further 72 h. MTT absorbance was read with a plate reader (Tecan Genios, Tecan US, Durham, NC, USA) at a wavelength of 495 nM. The blank value was subtracted from each measurement and the percentage of viability was determined by comparison with untreated cells. Apoptotic cells with a sub-diploid G1 content were quantitated by flow cytometry on samples that after treatment with drugs, were harvested, fixed in 70% ethanol, treated with RNAse and stained with propidiun iodide. Sub-G1 population was then revealed with a FACS-Scan (Becton Dickinson, San Jose, CA, USA).

### Western blot analysis

Cells were lysed in SDS/Tris-HCl and fractionated on SDS–PAGE. Proteins were blotted on PVDF membranes, saturated with non-fat dry milk and incubated overnight with the indicated primary antibody. After washing, the membranes were exposed to the appropriate secondary antibody for 1 h and finally the proteins of interest were detected by ECL (Pierce, Rockford, IL, USA).

### Recombinant protein purification

pET28 XIAP-Bir3 and XIAP-lkBir2Bir3 were used to produce the recombinant proteins for the binding assay (Mastrangelo *et al*, 2008). The plasmids were used to transform *Escherichia coli* strain BL21(DE3) and induced with 1 mM of IPTG. Bacteria grown in LB medium plus kanamycin and 50 *μ*M zinc acetate were harvested, resuspended in a buffer containing 50 mM Tris-HCl, pH 7.5, 200 mM NaCl and protease inhibitors, treated with 100 *μ*g ml^–1^ lysozyme for 30 min on ice, and then lysed by sonication. After elimination of debris by centrifugation, recombinant protein was purified using Ni-NTA (His-trap FFcrude, GE Healthcare), followed by gel filtration (Superdex 200, GE Healthcare). The recombinant proteins were eluted with 250 mM imidazole and thereafter stored in 20 mM Tris, pH 7.5, 200 mM NaCl and 10 mM DTT.

### Binding assays

Fluorescent polarisation-based binding assay was performed as described before (Mastrangelo *et al*, 2008; Cossu *et al*, 2009b). Briefly, two saturation curves were obtained by adding increasing concentrations of recombinant XIAP-BIR3 (241–356) or XIAP-lkBIR2BIR3 (124–356) to a fixed quantity of a fluorescent monomeric or dimeric SMAC-mimetic, respectively. The Kd was measured and used to determine the concentration of probe to be used in the competition experiment, in which SMAC-mimetics were added at rising concentrations. All data were plotted using Graphpad (San Diego, CA, USA).

### XIAP knock-down

XIAP knock-down was obtained by RNA interference using lentiviral vectors (Invitrogen, Carlsbad, CA, USA). Lentiviruses were produced in accordance to the manufacturer's instructions. In all, 293FT cells were transfected with ViraPower (Invitrogen) plus a pLenti vector carrying a sequence for XIAP RNA interference (Kashkar *et al*, 2007) or a control sequence (shLac), using Lipofectamine2000 (Invitrogen). The following day the medium was substituted and then collected after 24 h, clarified from cellular debris by centrifugation and added to the cells of interest. Transduced clones were selected by adding blasticidin to a final concentration of 6 *μ*g ml^–1^.

### Synthesis of SMAC-mimetics

The synthesis of monomeric SMAC067 has already been reported by us (Seneci *et al*, 2009), while the synthesis of the dimeric compounds SMAC074, SMAC075 and SMAC076 (Supplementary Figure S2) will be reported in a forthcoming paper (P Seneci, C Battaglia, L Belvisi, M Bolognesi, A Caprini, F Cossu, M de Matteo, D Delia, C Drago, D Lecis, L Manzoni, M Marizzoni, E Mastrangelo, M Milani, E Moroni, D Potenza, V Rizzo, F Servida, F Vasile and C Scolastico, 2010, to be submitted).

## Results

### TRAIL sensitivity in melanoma cell lines

**Figure 1:**
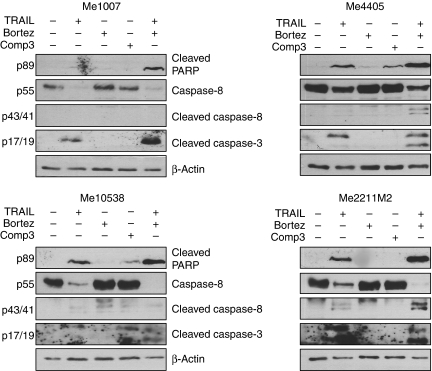
Sensitivity of melanoma cell lines to TRAIL, Bortezomib and SMAC-mimetics. Levels of cleaved PARP, caspase-3, caspase-8 or full-length caspase-8 in melanoma cells treated for 24 h with 50 ng ml^–1^ iz-TRAIL, 5 nM Bortezomib (4 nM in the case of Me1007) or 500 nM compound 3 or mock-treated. *β*-Actin is used as loading control.

We evaluated the sensitivity of the well-characterised melanoma cell lines Me4405, Me10538, Me2211M2 and Me1007 derived from patients of our institute, to increasing concentrations of iz-TRAIL. The proliferation of Me4405, Me10538 and Me2211M2 was inhibited by TRAIL in a dose-dependent manner, while Me1007 cells were resistant to this drug (Supplementary Figure S1) in keeping with the their low expression of caspase-8 (Zhang *et al*, 2001), a key mediator of the extrinsic apoptosis pathway (Chen *et al*, 2008). TRAIL-induced apoptosis was accompanied by cleavage of PARP, strongly detectable in all the lines except Me1007 (Figure 1), and by activation of caspase-8 yielding the cleaved p41 and 43 forms. These two bands were undetectable in Me1007 but evident in the other cell lines, and accompanied by the reduction of full-length caspase-8 (Figure 1). Caspase-8 cleaves and activates the effector caspase-3, whose cleaved forms were detectable in all lines tested after treatment with iz-TRAIL. We also analysed the response to Bortezomib and compound-3, a dimeric SMAC-mimetic (Li *et al*, 2004), but these agents had little effect, if any, in activating apoptosis at the doses used (Figure 1). Altogether, iz-TRAIL kills melanoma cells by activating the extrinsic apoptosis pathway involving caspase-8, -3 and PARP cleavage. This pathway is associated with caspase-8, whose faint levels in Me1007 preclude their responsiveness.

### Downregulation of XIAP is sufficient to restore TRAIL sensitivity even in melanoma cells with low expression of caspase-8

**Figure 2:**
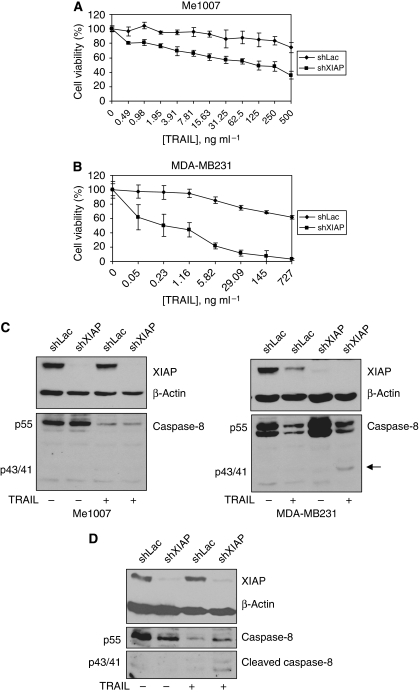
Role of XIAP in TRAIL resistance. Isogenic Me1007 (**A**) or MDA-MB231 (**B**) cell lines with normal (shLac) or downregulated levels of XIAP were treated with serial dilutions of iz-TRAIL. Cell proliferation was measured after 72 h by MTT assay. The figure is representative of three independent experiments carried out in triplicate (mean values±s.d.). (**C**) Levels of cleaved or full-length caspase-8 in Me1007 (left) or MDA-MB231 (right) with knock-down or normal levels of XIAP, treated or untreated with 50 ng ml^–1^ for 24 h. Arrow indicates the p43/41 forms of caspase-8. *β*-Actin is used as loading control. (**D**) Levels of caspase-8 and p43/41 caspase-8 in Me1007 with normal or knock-down XIAP, treated with 1 *μ*g ml^–1^ iz-TRAIL for 6 h.

As sensitisation of tumour cells to TRAIL can be achieved by downregulating XIAP (Vogler *et al*, 2008), we asked whether a similar response could be induced in Me1007 cells, in spite of their low expression of caspase-8. We therefore generated Me1007 cells stably expressing an shRNA-targeting XIAP or a control sequence (shLac) as negative control. Interestingly, XIAP knock-down restored TRAIL sensitivity in Me1007 (Figure 2A) and this was not cell-type specific because a similar phenomenon was observed in the mammary carcinoma cell line MDA-MB231 (Figure 2B). The treatment with 50 ng ml^–1^ iz-TRAIL for 24 h resulted in the accumulation of the p43/41 form of caspase-8 in MDA-MB231 (Figure 2C right) but not in Me1007 cells (Figure 2C left). Furthermore, this accumulation was evident in cells with downregulated levels of XIAP and not in control cells expressing the scrambled sequence (shLac, Figure 2C right). Interestingly, TRAIL treatment induced the downregulation of XIAP also in control cells. Cleaved caspase-8 was detectable in Me1007 cells after 6 h of treatment only in response to doses of iz-TRAIL⩾1 *μ*g ml^–1^, and even so the p43/41 form was evident only in cells with downregulated levels of XIAP (Figure 2D).

Thus, XIAP contributes to the resistance of melanoma cells to TRAIL and its downregulation favours the extrinsic apoptotic pathway.

### Bortezomib sensitises melanoma cells without affecting caspase-8 expression levels

**Figure 3:**
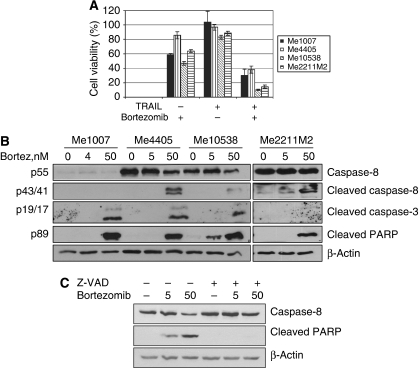
Bortezomib sensitises melanoma cells to TRAIL without affecting caspase-8 levels. (**A**) Cell proliferation evaluated 72 h after treatment with 5 nM Bortezomib (4 nM for Me1007) or 50 ng ml^–1^ of iz-TRAIL or a combination thereof (MTT assay). The figure is representative of three independent experiments carried out in triplicate (mean values±s.d.). (**B**) Levels of full-length caspase-8 or cleaved PARP, caspase-8 and -3 in lysates from melanoma cell lines treated with Bortezomib for 24 h at the indicated doses. (**C**) Levels of caspase-8 in lysates of Me1007 treated with Bortezomib for 24 h at the indicated doses (nM) and/or pretreated for 1 h with 15 *μ*M of z-VAD-fmk. Determination of PARP cleavage was used as a control to check activity of z-VAD-fmk and *β*-Actin served as loading control.

The proteasome inhibitor Bortezomib (Velcade/PS-341) is an effective drug in the treatment of multiple myeloma and other tumours, mainly functioning by inhibition of the NF-*κ*B survival pathway (reviewed by Boccadoro (Boccadoro *et al*, 2005)). Besides, it sensitises tumour cells to other agents, including TRAIL (Koschny *et al*, 2007). To determine whether Bortezomib potentiates the activity of TRAIL also in melanoma cells, we first tested it alone and found that at 4–5 nM it inhibits the growth of all melanoma cell lines. Significantly, the combination of TRAIL with Bortezomib markedly increased loss of cell viability in all melanoma lines (Figure 3A), paralleled by a rise in cleaved PARP (Figure 1).

Two inducers of endoplasmic reticulum (ER) stress, Thapsigargin and Tunicamycin, have been suggested to sensitise Me1007 to TRAIL by upregulation of TRAIL-R2 (Chen *et al*, 2007; Jiang *et al*, 2007) and caspase-8 (Chen *et al*, 2008). As Bortezomib can also cause ER stress (Boccadoro *et al*, 2005), we checked whether it upregulated caspase-8 in Me1007, but no evidence for this was observed, even in response to elevated doses of Bortezomib (Figure 3B). As the accumulation of procaspase-8 could be masked by its concomitant cleavage into the active forms, cells were pretreated with the pan caspase inhibitor z-VAD-fmk, which blocks caspase-8 cleavage. The treatment prevented caspase activation, as revealed by inhibition of PARP cleavage (Figure 3C), but did not affect the levels of caspase-8. Interestingly, treatment with Bortezomib at a concentration of 50 nM caused the accumulation of the p43/41 form of caspase-8 (Figure 3B). Altogether, these findings show that Bortezomib sensitises melanoma lines to TRAIL without affecting the caspase-8 levels.

### New SMAC-mimetics targeting the BIR2 and BIR3 domains of XIAP

Recently, we have described a group of 4-substituted azabicyclo[5.3.0]alkane SMAC-mimetics with submicromolar binding affinity for XIAP. We showed that the introduction of a new chemical arm in position 4 actually favoured the establishment of new interactions between the SMAC-mimetics and the BIR domains of XIAP (Mastrangelo *et al*, 2008; Cossu *et al*, 2009a), strengthening the binding of our compounds to the target protein. The SMAC-mimetic molecules were first modified by the introduction of a N-terminal methyl group to improve the cellular permeability (Sun *et al*, 2006), yielding SMAC067 (compound 4b, (Seneci *et al*, 2009)). As dimeric SMAC-mimetics have been shown to be more potent than their monomeric counterparts in activating apoptosis (Li *et al*, 2004), we synthesised three homodimeric compounds by coupling two SMAC067 units with different linkers (Supplementary Figure S2). The affinity of the compounds for XIAP BIR3 and lkBIR2/BIR3, measured by fluorescent polarisation as described (Nikolovska-Coleska *et al*, 2004, 2008) revealed that all three dimeric compounds had a lower affinity for BIR3 than the monomers (Table 1), suggesting that the linker region slightly hinders the interaction with BIR3 *in vitro*. However, the interaction of the homodimers with the lkBIR2/BIR3 was 10-fold higher compared with the monomer. Little differences in affinity could be detected between the three bivalent compounds towards BIR3 or lkBIR2/BIR3, with SMAC076 being the best interactor of both recombinant proteins.

**Figure 4:**
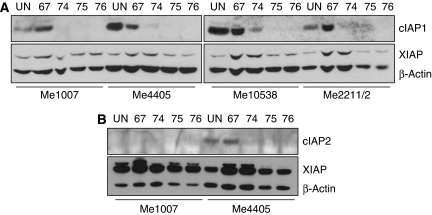
New SMAC-mimetics targeting IAPs. (**A**) Levels of cIAP1 and XIAP in melanoma cell lines treated for 3 h with 500 nM of the indicated compounds or mock-treated (UN). (**B**) Levels of cIAP2 and XIAP in melanoma cell lines treated for 3 h with 500 nM of the indicated compounds or mock-treated (UN). *β*-Actin served as loading control.

Aside from XIAP, SMAC-mimetics have been shown also to bind cIAP1 and cIAP2 (Varfolomeev *et al*, 2007; Vince *et al*, 2007). However, in contrast to XIAP, the binding of SMAC-mimetics to cIAPs induces their rapid auto-ubiquitination and subsequent proteasomal degradation. To determine whether the newly developed SMAC-mimetics caused the degradation of cIAP1 and cIAP2, we treated the four melanoma cell lines with the monomeric or dimeric SMAC-mimetics and analysed the effect of treatment on cIAP1/2 expression. Interestingly, all three dimeric compounds decreased the levels of cIAP1 and cIAP2 (Figures 4A and B and data not shown), most effectively in response to SMAC075 and SMAC076. SMAC067, on the contrary, had little effect, if any, on cIAP1 and cIAP2 levels. It is noteworthy that Me1007 cells were negative for the expression of cIAP2 (Figure 4B). Altogether, our new SMAC-mimetics not only bind XIAP with nanomolar affinities, but also induce the intracellular degradation of cIAP1 and cIAP2.

### SMAC-mimetics synergise with TRAIL

**Figure 5:**
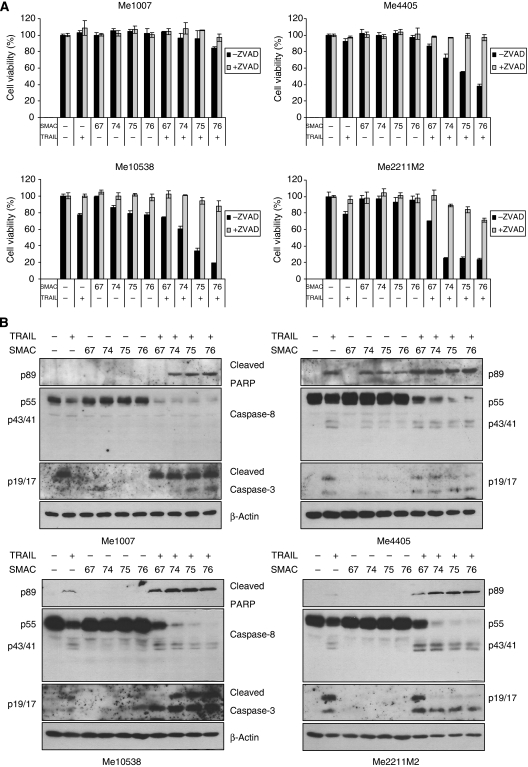
SMAC-mimetics synergise with TRAIL in triggering apoptosis. (**A**) Cell viability was evaluated 72 h after onset of treatment with 500 nM SMAC-mimetic and/or iz-TRAIL used at 50 ng ml^–1^ with or without pretreatment with 15 *μ*M of z-vad-fmk (MTT assay). The figure is representative of three independent experiments carried out in triplicate (mean values±s.d.). (**B**) Levels of caspase-8, cleaved PARP and caspase-3 in cells treated for 24 h with iz-TRAIL at 50 ng ml^–1^ and SMAC-mimetic at 500 nM. *β*-Actin served as loading control.

The *in vitro* synergism of SMAC-mimetics with TRAIL underscores the potential of this combination in cancer treatment (Li *et al*, 2004; Vogler *et al*, 2009). As a result of this, we analysed the synergism of TRAIL with our SMAC-mimetics more thoroughly. At the doses used here as single agents TRAIL and SMAC-mimetics had little effect on viability. However, in combination they potently induced loss of cell viability in all cell lines tested, except Me1007 (Figure 5A). This was associated with a strong activation of the apoptotic pathway, involving cleavage of caspase-8, activation of caspase-3 and accumulation of cleaved PARP (Figure 5B). Thus, cell death was due to apoptosis as this event was also abrogated by pretreatment with the pan-caspase inhibitor z-VAD-fmk (Figure 5A). Apoptosis was modest in Me1007, disappearance of full-length caspase-8 was not accompanied by accumulation of detectable p41/p43 of caspase-8 (Figure 5B). Nevertheless, a faint band of cleaved PARP appeared, suggesting that at least in some cells effector caspase activation had occurred. Western blot analysis confirmed the potent apoptogenic effect of iz-TRAIL in combination with SMAC-mimetics as compared with the single agents, and that the dimeric SMAC-mimetics are more powerful than the monomeric one in synergising with TRAIL (Figure 5B). Apoptotic cells were also assessed by flow cytometry analysis of the sub-G1 peak. The combined treatment of TRAIL with a SMAC-mimetic (SMAC075) augmented the proportion of the sub-diploid apoptotic population compared with single and mock treatments (Supplementary Table S3 and data not shown) in melanoma cells responsive to these drugs, while the increment was modest in Me1007.

It is noteworthy that the diverse linkers used have little effect on SMAC-mimetic activity, in accordance with the affinity for XIAP *in vitro* (Table 1) or stimulation of cIAP1/2 degradation (Figures 4A and B). Hence, our new SMAC-mimetics are able to inhibit melanoma cell growth by inducing apoptosis in synergism with iz-TRAIL.

### Bortezomib synergises with SMAC-mimetics in melanoma cell lines resistant to the combined effect of TRAIL/SMAC-mimetic

**Figure 6:**
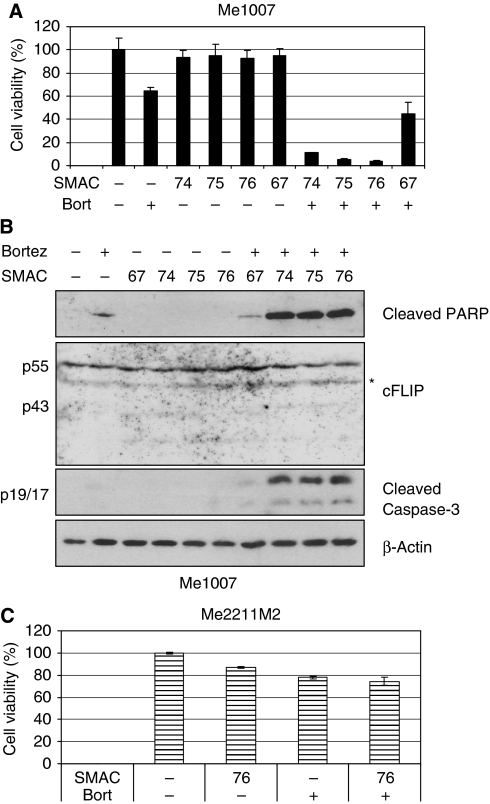
SMAC-mimetics synergise with Bortezomib in triggering apoptosis in Me1007 cells. (**A**) Me1007 cell viability was evaluated 72 h after treatment with 500 nM SMAC-mimetic and/or Bortezomib 4 nM (MTT assay). The figure is representative of three independent experiments in triplicate (mean values±s.d.). (**B**) Levels of cleaved PARP and caspase-3, and cFLIP in cells treated for 24 h with Bortezomib at 4 nM and SMAC-mimetics at 500 nM. *β*-Actin served as loading control. The asterisk indicates a nonspecific band. (**C**) Me2211M2 cell viability was evaluated 72 h after treatment with 500 nM SMAC076 and/or Bortezomib 5 nM (MTT assay). The figure is representative of two independent experiments carried out in triplicate (mean values±s.d.).

As Bortezomib sensitises cells to TRAIL-induced apoptosis at a different level than SMAC-mimetics, namely at the TRAIL DISC, we next addressed whether their combination would be synergistic in sensitising melanoma cells to TRAIL-induced apoptosis. We first tested the Me1007 cell line, which is poorly responsive to the TRAIL/SMAC-mimetics (Figure 5A), and found that already the combination of Bortezomib with dimeric SMAC-mimetics, induced almost complete loss of cell viability (Figure 6A). By contrast, the single compounds showed little activity (Figures 5A and 6A). The loss of cell viability by Bortezomib and SMAC-mimetics was due to apoptosis as it was accompanied by the accumulation of cleaved caspase-3 and PARP (Figure 6B), and to some extent dependent on caspase-8 because its depletion rescued cells from death triggered by Bortezomib (data not shown). Conversely, the cell lines sensitive to TRAIL/SMAC-mimetic were less sensitive to Bortezomib/SMAC-mimetic combination (Figure 6C and data not shown). As Bortezomib has been shown to be capable of modulating the expression levels of different proteins involved in regulating sensitivity to TRAIL (Koschny *et al*, 2007), we checked the expression of the caspase-8 inhibitor cFLIP. However, neither Bortezomib nor the SMAC-mimetics or their combinations affected cFLIP expression (Figure 6B).

These findings indicate that *in vitro* melanoma cells resistant to the combination of TRAIL with SMAC-mimetics can be killed by the combined effects of Bortezomib and SMAC-mimetic. Thus, the combination of SMAC-mimetics with Bortezomib, previously shown to kill multiple myeloma cells *in vitro* (Chauhan *et al*, 2007), may also be useful for the treatment of melanoma.

## Discussion

X-linked IAP is a pivotal anti-apoptotic regulator, owing to its capacity to bind and block both initiator and effector caspases (Deveraux *et al*, 1997). XIAP is overexpressed in many tumours in which it confers resistance to chemotherapeutic agents (Li *et al*, 2004; Oost *et al*, 2004) and to TRAIL, an apoptosis-inducing biotherapeutic drug (Walczak *et al*, 1999) currently tested in various clinical trials (Ashkenazi and Herbst, 2008; Newsom-Davis *et al*, 2009). Unlike the other apoptosis-inducing ligands of the TNF superfamily, TNF and CD95L, TRAIL is not toxic for normal cells, suggesting its potential exploitability for treatment purposes. In spite of the fact that different members of the IAP family show high homology and can have redundant roles, nevertheless XIAP is so far the only member known to affect sensitivity to TRAIL-induced apoptosis (Cummins *et al*, 2004), providing a rationale for combining TRAIL with drugs targeting XIAP.

In our work, we first treated some melanoma cell lines with iz-TRAIL, a recombinant TRAIL fused with a synthetic isoleucine zipper that favours trimerisation, thereby enhancing its activity (Ganten *et al*, 2006), a phenomenon we had first reported for a different version of TRAIL together with an *in vivo* half-life of approximately 1 h (Walczak *et al*, 1997). Together, increased activity because of stable trimer formation and increased half-life renders isoleucine zipper tagged forms of TRAIL potentially better drug candidates as we previously showed (Walczak *et al*, 1999; Ganten *et al*, 2004, 2006) and has recently been confirmed by others (Rozanov *et al*, 2009). Three melanoma lines were sensitive to iz-TRAIL at high concentrations but their response could be amplified by concomitant administration of SMAC-mimetics. By contrast and in concordance with a previous report (Zhang *et al*, 2001), the fourth line tested, Me1007, was resistant to iz-TRAIL treatment, most likely because of caspase-8 expression being too low to enable activation of the extrinsic apoptosis pathway. Interestingly, the knock-down of XIAP and, partially, the simultaneous administration of the new SMAC-mimetic compounds we developed, could restore sensitivity of Me1007 cells to TRAIL-induced apoptosis. In these cells, low levels of caspase-8 could probably activate little caspase-3 that XIAP keeps in check preventing the apoptosis cascade, but when XIAP is targeted, the levels of caspase-3 might become enough to trigger apoptosis.

Interestingly, in presence of downregulated levels of XIAP, there is an accumulation of the cleaved p43 and p41 forms of caspase-8, both in melanoma and mammary carcinoma cell lines. XIAP could influence this event in two ways, directly by ubiquitinating the cleaved form of caspase-8 and causing its degradation, or indirectly by blocking caspase-3, which was previously shown to cleave caspase-8 (Wieder *et al*, 2001; Lee *et al*, 2005; Hayakawa *et al*, 2008). As XIAP was shown to interact with caspases 3, 7 and 9, but not 8 (Deveraux *et al*, 1998), it is unlikely that the accumulation of the p43/41 forms of caspase-8 is caused directly by XIAP. More likely, XIAP, by inhibiting caspase-3, prevents its positive feedback loop, by which caspase-8 is further cleaved (Wieder *et al*, 2001; Lee *et al*, 2005; Hayakawa *et al*, 2008). Thus, lower levels of XIAP allow the accumulation of the cleaved form of caspase-8.

SMAC-mimetics have been shown to activate apoptosis also as stand-alones in a few cell lines (Petersen *et al*, 2007). This mechanism is independent from their activity on XIAP, and rather because of their capacity of targeting cIAP1 and cIAP2 (Varfolomeev *et al*, 2007; Vince *et al*, 2007). In this case, SMAC-mimetics do not trigger apoptosis by displacing caspases from XIAP, but by inducing TNF and rendering cancer cells sensitive to autocrine TNFα (Varfolomeev *et al*, 2007; Vince *et al*, 2007; Bertrand *et al*, 2008). According to this, the apoptotic pathway depends on caspase-8 presence, but is not influenced by XIAP levels (Gaither *et al*, 2007). Indeed, the new dimeric SMAC-mimetics we present here are able to trigger rapid and complete degradation of cIAP1, and, consequently, to cause apoptosis in MDA-MB231 cells as single agents (data not shown).

Nevertheless, SMAC-mimetics are more widely utilisable in association with other drugs to favour their activity (Li *et al*, 2004). In this study, we show that the newly developed SMAC-mimetics can facilitate the apoptotic cascade stimulated by TRAIL, rendering melanoma cells, which are notoriously resistant to chemotherapy, more sensitive to this ligand. Interestingly, the Me1007 cell line, resistant to SMAC-mimetic/TRAIL, became extremely sensitive to the combination of SMAC-mimetics and Bortezomib, the latter widely used in cancer therapy. This observation raises the possibility that SMAC-mimetics could be useful in combination with diverse drugs.

In conclusion, SMAC-mimetics targeting members of the IAP family, especially XIAP, offer a new strategy for the treatment of chemotherapy-resistant melanomas, particularly when used in combination with other drugs like Bortezomib, TRAIL or TRAIL-R agonists.

## Figures and Tables

**Table 1:** Affinities of newly developed SMAC-mimetics for XIAP BIR3 and lkBIR2BIR3

**Compound**	**IC_50_ BIR3±s.e.% nM**	**IC_50_ lkBIR23±s.e.% nM**
67^a^	104±14.2	54±48.5
74	180.5±13.2	5.91±22.2
75	178.8±15.9	3.18±18.4
76	157±13.5	2.27±24.5

Abbreviations: BIR=baculoviral inhibitor repeats; SMAC=second mitochondria-derived activator of caspase; s.e.%=standard error % XIAP=X-linked inhibitor of apoptosis protein.

The affinities of the new SMAC-mimetic compounds were evaluated by a fluorescent polarisation-based binding assay using increasing concentrations of SMAC-mimetics, two fluorescent dyes and the recombinant proteins containing the BIR3 or the lkBIR2BIR3 domains of XIAP as described in Material and Methods.

a See Seneci *et al* (2009).
