# Supplementary figures and images for: Novel SMAC-mimetics synergistically stimulate melanoma cell death in combination with TRAIL and Bortezomib
Source: Br J Cancer. 2010 May 11;102(12):1707–16. doi: 10.1038/sj.bjc.6605687 (PMC2883696; doi:10.1038/sj.bjc.6605687)

## SUPPLEMENTAL DATA

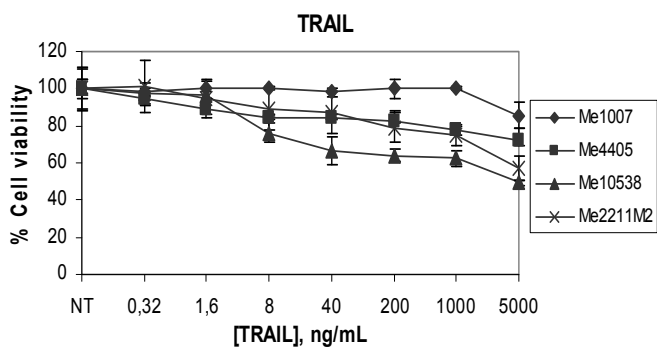

# S1

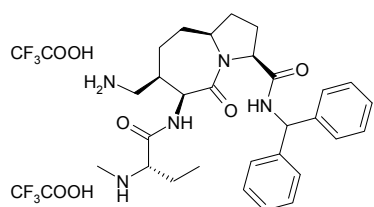

SMAC067

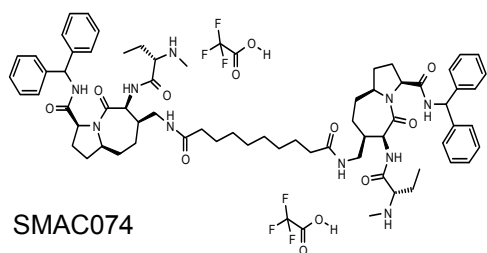

SMAC074

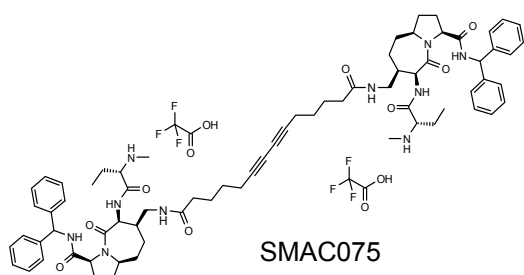

SMAC075

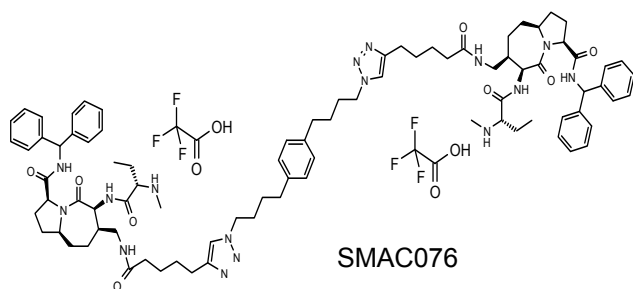

SMAC076

**S2**

Supplement: Supplementary Data [file 6605687x1.pdf]
