# Supplementary material for: Novel SMAC-mimetics synergistically stimulate melanoma cell death in combination with TRAIL and Bortezomib
Source: Br J Cancer. 2010 May 11;102(12):1707–16. doi: 10.1038/sj.bjc.6605687 (PMC2883696; doi:10.1038/sj.bjc.6605687)
Supplement: Supplementary Information [file 6605687x2.doc]

**Supplementary information**

**S1** Sensitivity of melanoma cell lines to TRAIL. Cell viability was evaluated 72 h after treatment with serial dilutions of iz-TRAIL (MTT assay). The figure is representative of 3 independent experiments done in triplicate (mean values±SD).

**S2** Molecular structures of our new compounds. SMAC074, SMAC075 and SMAC076 are homodimers obtained by tethering two molecules of the monomeric SMAC-mimetic SMAC067 with three different linkers.

**S3** Analysis of the sub-G1 population of melanoma cell lines treated 96 hrs with, 50 ng/mL of iz-TRAIL and/or 500nM SMAC075, or mock treated (UN).

|  | **Treatment** | **%**  **Apoptosis** |
| --- | --- | --- |
| Me1007 | UN | 1.7 |
|  | SMAC075 | 2.0 |
|  | TRAIL | 2.2 |
|  | SMAC075+TRAIL | 7.8 |
| Me4405 | UN | 6.5 |
|  | SMAC075 | 11.1 |
|  | TRAIL | 25.8 |
|  | SMAC075+TRAIL | 35.1 |
| Me10538 | UN | 2.9 |
|  | SMAC075 | 4.9 |
|  | TRAIL | 5.5 |
|  | SMAC075+TRAIL | 11.3 |
| Me2211M2 | UN | 3.2 |
|  | SMAC075 | 3.5 |
|  | TRAIL | 30.0 |
|  | SMAC075+TRAIL | 44.4 |

*Characterization of Smac-mimetics compounds*

^1^H-NMR spectra were recorded on Bruker Avance in CDCl_3_, CD_3_OD or D_2_O as solvent at 400 or 600 MHz. ^13^C-NMR spectra were recorded in CDCl_3_, CD_3_OD or D_2_O as solvent at 100 or 125 MHz. Coupling constants are given in hertz and are rounded to the nearest 0.1 Hz. LC-MS data were collected with an Agilent 1100 HPLC connected to a Bruker Esquire 3000+ ion trap mass spectrometer through an ES interface.

**SMAC067.** MW 733.7, bis-trifluoroacetate salt, amorphous white solid. *Analytical characterization*: *^1^H-NMR* (400 MHz, D_2_O): δ: 7.40-7.21 (m, 10H), 5.70 (s, 1H), 4.65-4.61 (m, 1H), 4.52-4.43 (m, 1H), 4.09-3.98 (m, 1H), 3.94-3.85 (m, 1H), 3.14-3.05 (d, J = 12.0 Hz, 1H), 2.91-2.80 (dd, J = 12.0, 11.2 Hz, 1H), 2.62 (s, 3H), 2.24-1.79 (m, 8H), 1.78-1.53 (m, 3H), 0.88 (t, J = 6.4 Hz, 3H); *^13^C-NMR* (100.6 MHz, D_2_O): δ: 172.9, 169.2, 169.0, 140.9, 140.7, 129.0, 128.9, 127.8, 127.4, 127.2, 62.6, 61.9, 58.4, 57.8, 54.3, 40.7, 36.1, 32.2, 31.5, 29.9, 28.3, 28.1, 23.3, 8.1. ESI-MS: m/z 506.3 [M+H]^+^, 528.3 [M+Na]^+^.

**SMAC074.** MW 1405.59, bis-trifluoroacetate salt, amorphous white solid. *Analytical characterization:* *^1^H-NMR* (400 MHz, D_2_O): δ: 8.80 (m, 2H), 7.40-7.20 (m, 20H), 6.14 (s, 2H), 4.67 (m, 4H), 4.00 (m, 2H), 3.87 (bs, 2H), 3.44 (d, J = 13.6 Hz, 2H), 3.01 (t, J = 10 Hz, 2H), 2.70 (s, 6H), 2.85-2.70 (m, 6H), 2.70-1.75 (m, 16H), 1.70-1.55 (m, 8H), 1.32 (s, 8H), 1.05 (d, J = 7.0 Hz, 6H); *^13^C-NMR* (100 MHz, D_2_O): δ: 175.1, 171.6, 169.6, 167.5, 141.7, 141.5, 128.3, 128.1, 127.2, 127.1, 126.9, 62.6, 61.5, 58.2, 57.0, 54.3, 40.9, 38.2, 35.7, 32.7, 31.7, 31.0, 30.0, 28.9, 27.5, 25.6, 23.3, 8.0. ESI-MS: m/z 1177.6 [M+H]^+^, 589.2 [M+2H]^2+^.

**SMAC075.** MW 1453.64, bis-trifluoroacetate salt, amorphous white solid. *Analytical characterization:* *^1^H-NMR* (400 MHz, D_2_O): δ: 8.80 (m, 2H), 7.40-7.20 (m, 20H), 6.14 (s, 2H), 4.67 (m, 4H), 4.02 (bs, 2H), 3.87 (bs, 2H), 3.44 (d, J = 13.2 Hz, 2H), 3.07 (t, J = 11.2 Hz, 2H), 2.72 (s, 6H), 2.40-2.20 (m, 10H), 2.20-180 (m, 16H), 1.75-1.60 (m, 8H), 1.60-1.45 (m, 4H), 1.05 (d, J = 7.1 Hz, 6H); *^13^C-NMR* (100 MHz, D_2_O): δ: 174.6, 171.6, 169.6, 167.5, 141.7, 141.5, 128.3, 128.1, 127.3, 127.2, 127.0, 126.9, 76.3, 65.4, 62.6, 61.6, 58.3, 57.0, 54.3, 40.9, 38.3, 35.1, 32.7, 31.7, 31.0, 30.2, 27.7, 27.5, 24.8, 23.2, 18.1, 7.8. ESI-MS: m/z 1226.0 [M+H]^+^, 613.7 [M+2H]^2+^.

**SMAC076.** MW 1728.01, bis-trifluoroacetate salt, amorphous white solid. *Analytical characterization:* *^1^H-NMR* (400 MHz, D_2_O): δ: 8.80 (m, 2H), 7.76 (s, 2H), 7.40-7.15 (m, 20H), 7.06 (s, 4H), 6.14 (s, 2H), 4.66 (m, 4H), 4.37 (t, J = 6.4 Hz, 4H), 4.00 (m, 2H), 3.87 (m, 2H), 3.44 (d, J = 13.2 Hz, 2H), 3.05 (t, J = 11.6 Hz, 2H), 2-74-2.68 (m, 10H), 2.59 (t, J = 6.8 Hz, 4H), 2.26 (m, 6H), 2.15-1.75 (m, 21H), 1.70-1.50 (m, 17H), 1.06 (t, J = 7.2 Hz, 6H); *^13^C-NMR* (100 MHz, D_2_O): δ: 174.7, 171.6, 169.6, 167.5, 147.1, 141.7, 141.6, 139.2, 128.3, 128.1, 127.3, 127.1, 126.9, 122.2, 62.6, 61.6, 58.3, 57.1, 54.3, 50.0, 41.0, 38.3, 35.3, 34.2, 32.7, 31.7, 31.0, 30.2, 29.3, 28.5, 28.0, 27.6, 25.0, 24.3, 23.2, 7.8. ESI-MS: m/z 1500.2 [M+H]^+^, 751.2 [M+2H]^2+^.

**Footnotes**

† P. Seneci, C. Battaglia, L. Belvisi, M. Bolognesi, A. Caprini, F. Cossu, M. de Matteo, D. Delia, C. Drago, D. Lecis, L. Manzoni, M. Marizzoni, E. Mastrangelo, M. Milani, E. Moroni, D. Potenza, V. Rizzo, F. Servida, F. Vasile and Carlo Scolastico, *Bioorg. Med. Chem*, to be submitted, 2009.
